# Supplementary material for: A Time Series Analysis Evaluating Antibiotic Prescription Rates in Long-Term Care during the COVID-19 Pandemic in Alberta and Ontario, Canada
Source: Antibiotics (Basel). 2022 Jul 26;11(8):1001. doi: 10.3390/antibiotics11081001 (PMC9330385; doi:10.3390/antibiotics11081001)
Supplement: Supplementary file 1 [file antibiotics-11-01001-s001.zip › antibiotics-1829504-supplementary.pdf]

Supplementary Table S1. Major classes of antibiotics using the Anatomical Therapeutic Chemical (ATC) classification, WHO AWaRe classification and most common indication for use

Class	Oral antibiotics	WHO AWaRe classification*	Most common indication for use#
**J01A – Tetracyclines**	J01AA01 - Demeclocycline	Watch	Other
	J01AA02 - Doxycycline	Access	
	J01AA07 - Tetracycline	Access	
	J01AA08 - Minocycline	Watch	
**J01C – Beta-lactam antibacterials, penicillins**	J01CA01 - Ampicillin	Access	Respiratory tract infections
	J01CA04 - Amoxicillin	Access	
	J01CE02 - Phenoxymethylpenicillin	Access	
	J01CF02 - Cloxacillin	Access	
	J01CR02 - Amoxicillin and enzyme inhibitor	Access	
**J01D – Other beta-lactam antibacterials**	J01DB01 - Cephalexin	Access	Skin and soft tissue infections
	J01DB05 - Cefadroxil	Access	
	J01DC02 - Cefuroxime	Watch	Respiratory tract infections
	J01DC04 - Cefaclor	Watch	
	J01DC10 - Cefprozil	Watch	
	J01DD08 - Cefixime	Watch	
**J01E – Sulfonamides and Trimethoprim**	J01EA01 - Trimethoprim	Access	Urinary tract infections
	J01EE01 - Sulfamethoxazole and trimethoprim	Access	
**J01F – Macrolides, Lincosamides and Streptogramins**	J01FA01 - Erytromycin	Watch	Respiratory tract infections
	J01FA02 - Spiramycin	Watch	
	J01FA09 - Clarithromycin	Watch	
	J01FA10 - Azithromycin	Watch	
	J01FF01 - Clindamycin	Access	Skin and soft tissue infections
**J01M – Quinolone antibacterials**	J01MA01 - Ofloxacin	Watch	Urinary tract infections
	J01MA02 - Ciprofloxacin	Watch	
	J01MA06 - Norfloxacin	Watch	
	J01MA12 - Levofloxacin	Watch	Respiratory tract infections
	J01MA14 - Moxifloxacin	Watch	
**J01X – Other antibacterials**	J01XC01 - Fusidic acid	Watch	Other
	J01XD01 - Metronidazole	Access	
	J01XE01 - Nitrofurantoin	Access	
	J01XX01 - Fosfomycin	Watch	
	J01XX08 - Linezolid	Reserve	

* 2021 AWaRe classification: WHO access, watch, reserve, classification of antibiotics for evaluation and monitoring of use. WHO reference number: WHO/HMP/HPS/EML/2021.04

Adapted from Schwartz KL, Achonu C, Brown KA, et al. Regional variability in outpatient antibiotic use in Ontario, Canada: a retrospective cross-sectional study. CMAJ Open 2018;6:E445–52. doi:10.9778/CMAJO.20180017

Supplementary Table S2. Sensitivity analysis 1 – Interrupted time series analysis showing the change in weekly oral antibiotic prescription rate per 1,000 LTCF residents after March 2020 in long-term care facilities in Alberta and Ontario, Canada; modeling a gradual increase of the step function in 2 weeks (week 10 and 11 of 2020)

		Alberta							Ontario						
**Prescription rate category**		**Model para-meters ***	**Step change**	**95% CI**	**p-value**	**Slope change**	**95% CI**	**p-value**	**Model para-meters ***	**Step change**	**95% CI**	**p-value**	**Slope change**	**95% CI**	**p-value**
**Overall**		(0,1,2) (1,0,0)[52]	-3.6526	-6.5143 – -0.7908	**0.012**	0.0166	-0.1117 – 0.1449	0.800	(0,1,1) (0,1,1)[52]	-1.2889	-3.6701 – 1.0923	0.289	0.0167	-0.1004 – 0.1338	0.780
**Sex**	**Females**	(1,1,1) (0,0,1)[52]	-3.6470	-6.7484 – -0.5457	**0.021**	0.0074	-0.0986 – 0.1135	0.891	(0,1,1) (0,1,1)[52]	-1.4341	-4.0618 – 1.1936	0.285	0.0220	-0.1089 – 0.1529	0.742
	**Males**	(1,0,1) (1,0,0)[52]	-2.0920	-5.1167 – 0.9327	0.175	0.0380	-0.0712 – 0.1473	0.495	(0,1,1) (0,1,1)[52]	-1.2521	-3.6842 – 1.1800	0.313	0.0120	-0.0799 – 0.1039	0.798
**Age**	**65-69**	ARIMA (0,0,0)	0.0461	-2.8305 – 2.9226	0.975	0.0462	-0.0601 – 0.1525	0.395	ARIMA (2,0,3)	1.7147	-2.5466 – 5.9759	0.430	-0.1415	-0.3091 – 0.0261	0.130
	**70-74**	ARIMA (0,0,5)	-2.0434	-5.3127 – 1.2260	0.221	0.0794	-0.0408 – 0.1996	0.196	(0,1,1) (1,0,0)[52]	-2.1006	-4.7971 – 0.5959	0.127	-0.0032	-0.1051 – 0.0988	0.951
	**75-79**	ARIMA (0,1,1)	-1.6438	-4.8179 – 1.5302	0.310	-0.0977	-0.2104 – 0.0149	0.089	(0,1,2) (1,0,0)[52]	-3.5209	-6.3478 – -0.6940	**0.015**	0.0089	-0.0969 – 0.1147	0.869
	**80-84**	(0,1,1) (1,0,0)[52]	-3.6036	-7.1481 – -0.0592	**0.046**	-0.0191	-0.1516 – 0.1134	0.777	(0,1,1) (0,1,1)[52]	-2.0959	-4.4720 – 0.2803	0.084	-0.0112	-0.1032 – 0.0808	0.811
	**85-89**	ARIMA (1,0,1)	-3.0476	-6.4634 – 0.3682	0.080	0.0474	-0.0770 – 0.1718	0.455	(0,1,1) (1,0,0)[52]	-1.0162	-4.8450 – 2.8126	0.603	0.0084	-0.2070 – 0.2237	0.939
	**90+**	(1,0,1) (1,0,0)[52]	-1.6776	-5.3246 – 1.9694	0.367	0.0309	-0.0986 – 0.1603	0.640	(0,1,1) (0,1,1)[52]	-0.6929	-3.5748 – 2.1890	0.637	0.0232	-0.1129 – 0.1594	0.738
**ATC Class**	**J01A – Tetracyclines**	ARIMA (1,0,1)	0.0198	-0.5600 – 0.5996	0.947	-0.0019	-0.0231 – 0.0192	0.857	(0,1,2) (1,0,0)[52]	-0.1568	-0.3380 – 0.0244	0.090	-0.0071	-0.0143 – 0.0002	0.057
	**J01C – Beta-lactams**	(1,1,2) (0,0,1)[52]	-1.2648	-2.1564 – -0.3732	**0.005**	0.0095	-0.0194 – 0.0384	0.519	(0,1,1) (0,1,1)[52]	-1.0490	-1.7486 – -0.3494	**0.003**	0.0056	-0.0216 – 0.0328	0.687
	**J01D – Other beta-lactams**	ARIMA (0,1,1)	-0.8172	-1.4639 – -0.1706	**0.013**	0.0150	-0.0072 – 0.0372	0.185	(1,1,3) (1,0,0)[52]	-0.7221	-1.1664 – -0.2777	**0.001**	0.0061	-0.0158 – 0.0280	0.588

	**J01E – Sulfonamides and trimethoprim**	ARIMA (0,0,0)	0.2613	-0.0659 – 0.5885	0.118	-0.0125	-0.0246 – -0.0004	**0.042**	(0,1,1) (0,0,1)[52]	0.2651	-0.0274 – 0.5576	0.076	0.0030	-0.0068 – 0.0128	0.546
	**J01F – Macrolides, lincosamides and streptogramins**	(1,0,1) (0,0,1)[52]	-0.1959	-0.5236 – 0.1318	0.241	0.0038	-0.0081 – 0.0157	0.532	(0,1,1) (1,0,0)[52]	0.3795	-0.2521 – 1.0111	0.239	-0.0175	-0.0592 – 0.0242	0.410
	**J01M – Quinolones**	(1,0,1) (0,0,1)[52]	-0.5902	-1.2351 – 0.0547	0.073	0.0038	-0.0214 – 0.0252	0.873	(0,1,1) (1,1,0)[52]	0.4008	-0.7070 – 1.5086	0.478	0.0059	-0.0571 – 0.0689	0.854
	**J01X – Other antibacterials**	ARIMA (0,0,2)	0.2704	-0.0759 – 0.6167	0.126	0.0011	-0.0158 – 0.0139	0.860	(1,0,1) (1,0,0)[52]	0.3651	-0.1190 – 0.8493	0.139	0.0107	-0.0053 – 0.0267	0.190
**Individual antibiotics**	**Amoxicillin**	(1,1,2) (0,0,1)[52]	-0.6254	-1.0672 – -0.1835	**0.006**	0.0036	-0.0114 – 0.0185	0.639	(0,1,2) (1,0,1)[52]	-0.7425	-1.0118 – -0.4732	**<0.001**	0.0094	-0.0009 – 0.0197	0.075
	**Amoxicillin/ clavulanic acid**	ARIMA (1,0,1)	-0.3719	-0.9810 – 0.2372	0.231	0.0036	-0.0186 – 0.0258	0.752	(0,1,1) (1,1,0)[52]	0.0145	-0.6100 – 0.6391	0.964	-0.0163	-0.0468 – 0.0143	0.298
	**Azithromycin**	(1,0,1) (0,0,1)[52]	-0.2325	-0.5653 – 0.1003	0.171	0.0066	-0.0055 – 0.0188	0.284	(0,1,1) (0,1,1)[52]	0.6010	0.1096 – 1.0924	**0.017**	-0.0160	-0.0455 – 0.0135	0.289
	**Cephalexin**	ARIMA (0,1,1)	-0.6144	-1.1316 – -0.0973	**0.020**	0.0106	-0.0065 – 0.0277	0.224	(2,0,2) (1,0,0)[52]	-0.3916	-0.7239 – -0.0593	**0.021**	0.0119	0.0002 – 0.0236	**0.046**
	**Clarithromycin**								ARIMA (2,1,3)	-0.0038	-0.1190 – 0.1114	0.948	-0.0029	-0.0090 – 0.0032	0.345
	**Doxycycline**	ARIMA (1,1,1)	-0.2101	-0.8622 – 0.4420	0.528	-0.0011	-0.0314 – 0.0293	0.945	(1,1,1) (0,0,1)[52]	-0.1657	-0.3232 – -0.0082	**0.039**	-0.0074	-0.0135 – -0.0014	**0.016**
	**Fosfomycin**								(0,1,1) (1,0,0)[52]	0.0141	-0.2451 – 0.2733	0.915	-0.0058	-0.0159 – 0.0043	0.263
	**Nitrofurantoin**	ARIMA (2,0,2)	0.3356	0.0277 – 0.6435	**0.033**	-0.0045	-0.0159 – 0.0068	0.432	(0,1,1) (1,0,0)[52]	-0.0438	-0.4398 – 0.3522	0.828	0.0135	-0.0015 – 0.0285	0.078
	**Penicillin**								ARIMA (0,1,1)	-0.0348	-0.0623 – -0.0073	**0.013**	0.0009	- 0.0001 – 0.0018	0.077

Bold: p-value < 0.05

* Model parameters are displayed as 'SARIMA (p,d,q) (P,D,Q)S'. If no seasonality was present, the model is presented as: ARIMA(p,d,q)

No results are shown for clarithromycin, fosfomycin and penicillin in Alberta to preserve subject anonymity, as the number of dispensations was on average less than 5 per week

LTCF: Long-term care facility; SARIMA: seasonal autoregressive integrated moving average; ARIMA: autoregressive integrated moving average; 95% CI: 95% Confidence interval;
ATC: Anatomical Therapeutic Chemical Classification

Supplementary Table S3. Sensitivity analysis 2 – Interrupted time series analysis showing the change in weekly oral antibiotic prescription rate per 1,000 LTCF residents after March 2020 in long-term care facilities in Alberta and Ontario, Canada; modeling a sudden increase of the step function in week 10 of 2020

		Alberta							Ontario						
**Prescription rate category**		**Model para-meters ***	**Step change**	**95% CI**	**p-value**	**Slope change**	**95% CI**	**p-value**	**Model para-meters ***	**Step change**	**95% CI**	**p-value**	**Slope change**	**95% CI**	**p-value**
**Overall**		(0,1,2) (1,0,0)[52]	-3.0048	-5.6099 – -0.3998	**0.024**	0.0003	-0.1276 – 0.1281	0.997	(0,1,1) (0,1,1)[52]	-0,3289	-2.5284 – 1.8706	0.769	-0.0005	-0.1198 – 0.1189	0.994
**Sex**	**Females**	(1,1,1) (0,0,1)[52]	-3.0499	-5.8147 – -0.2851	**0.031**	-0.0069	-0.1050 – 0.1281	0.890	(0,1,1) (0,1,1)[52]	-0.2921	-2.7141 – 2.1300	0.813	0.0016	-0.1321 – 0.1353	0.981
	**Males**	(1,0,1) (1,0,0)[52]	-1.6764	-4.4224 – 1.0696	0.231	0.0252	-0.0771 – 0.1276	0.629	(0,1,1) (0,1,1)[52]	-0.7303	-3.0883 – 1.6278	0.544	0.0022	-0.0923 – 0.0967	0.964
**Age**	**65-69**	ARIMA (0,0,0)	-0.0018	-2.6932 – 2.6896	0.999	0.0477	-0.0533 – 0.1487	0.354	(1,0,2) (1,0,0)[52]	-0.4722	-4.1175 – 3.1732	0.800	-0.0749	-0.2094 – 0.0596	0.275
	**70-74**	ARIMA (0,0,5)	-1.3519	-4.4327 – 1.7290	0.390	0.0575	-0.0575 – 0.1725	0.327	(0,1,1) (1,0,0)[52]	-1.8079	-4.3234 – 0.7076	0.159	-0.0112	-0.1101 – 0.0878	0.825
	**75-79**	ARIMA (0,1,1)	-1.0712	-4.0428 – 1.9005	0.480	-0.1117	-0.2199 – -0.0034	**0.043**	(0,1,2) (1,0,0)[52]	-3.1005	-5.7110 – -0.4901	**0.020**	-0.0036	-0.1074 – 0.1001	0.945
	**80-84**	(0,1,1) (1,0,0)[52]	-3.6762	-7.0230 – -0.3294	**0.031**	-0.0230	-0.1533 – 0.1073	0.730	(0,1,1) (1,1,0)[52]	-1.5831	-3.9175 – 0.7513	0.184	-0.0225	-0.1166 – 0.0717	0.640
	**85-89**	ARIMA (1,0,1)	-2.8152	-5.9635 – 0.3332	0.080	0.0399	-0.0776 – 0.1574	0.506	(0,1,1) (1,0,0)[52]	-0.0007	-3.3466 – 3.3453	1.000	-0.0096	-0.2250 – 0.2057	0.930
	**90+**	(1,0,1) (1,0,0)[52]	-1.1406	-4.1701 – 1.8889	0.461	0.0139	-0.0987 – 0.1264	0.809	(0,1,1) (0,1,1)[52]	0.3001	-2.3549 – 2.9552	0.825	0.0069	-0.1300 – 0.1438	0.921
**ATC Class**	**J01A – Tetracyclines**	ARIMA (1,0,1)	0.0394	-0.4626 – 0.5413	0.878	-0.0025	-0.0217 – 0.0167	0.796	(1,1,1) (0,0,1)[52]	-0.2060	-0.3570 – -0.0550	**0.007**	-0.0070	-0.0132 – -0.0009	**0.025**
	**J01C – Beta-lactams**	(1,1,2) (0,0,1)[52]	-0.8744	-1.7869 – 0.0382	0.060	0.0008	-0.0273 – 0.0289	0.954	(0,1,1) (0,1,1)[52]	-0.3872	-1.2614 – 0.4869	0.385	-0.0073	-0.0446 – 0.0300	0.702
	**J01D – Other beta-lactams**	ARIMA (0,1,1)	-0.6740	-1.2579 – -0.0901	**0.024**	0.0115	-0.0096 – 0.0326	0.284	(1,1,3) (1,0,0)[52]	-0.5397	-0.9390 – -0.1404	**0.008**	0.0020	-0.0191 – 0.0231	0.851

	**J01E – Sulfonamides and trimethoprim**	ARIMA (0,0,0)	0.2355	-0.0708 – 0.5417	0.132	-0.0117	-0.0232 – -0.0002	**0.047**	(0,1,1) (0,0,1)[52]	0.2560	-0.0200 – 0.5320	0.069	0.0035	-0.0061 – 0.0131	0.475
	**J01F – Macrolides, lincosamides and streptogramins**	(1,0,1) (0,0,1)[52]	-0.1464	-0.4465 – 0.1537	0.339	0.0023	-0.0089 – 0.0134	0.688	(0,1,1) (0,1,1)[52]	0.4773	0.0354 – 0.9193	**0.034**	-0.0116	-0.0371 – 0.0138	0.369
	**J01M – Quinolones**	(1,0,1) (0,0,1)[52]	-0.4794	-1.0782 – 0.1195	0.117	-0.0015	-0.0236 – 0.0206	0.894	(0,1,1) (1,1,0)[52]	0.4851	-0.4307 – 1.4009	0.299	0.0058	-0.0553 – 0.0669	0.852
	**J01X – Other antibacterials**	ARIMA (0,1,3)	0.0861	-0.2955 – 0.4677	0.658	0.0029	-0.0096 – 0.0155	0.645	(1,0,1) (1,0,0)[52]	0.3549	-0.0761 – 0.7859	0.107	0.0110	-0.0037 – 0.0257	0.141
**Individual antibiotics**	**Amoxicillin**	(1,1,2) (0,0,1)[52]	-0.5675	-0.9690 – -0.1659	**0.006**	0.0017	-0.0122 – 0.0157	0.810	(0,1,2) (1,0,1)[52]	-0.5368	-0.8230 – -0.2505	**<0.001**	0.0045	-0.0063 – 0.0153	0.414
	**Amoxicillin/ clavulanic acid**	ARIMA (1,0,1)	-0.1009	-0.6766 – 0.4748	0.731	-0.0047	-0.0262 – 0.0167	0.665	(0,1,1) (1,1,0)[52]	0.0447	-0.4800 – 0.5694	0.867	-0.0167	-0.0465 – 0.0131	0.273
	**Azithromycin**	(1,0,1) (0,0,1)[52]	-0.1556	-0.4524 – 0.1412	0.304	0.0044	-0.0069 – 0.0156	0.446	(0,1,1) (0,1,1)[52]	0.4225	0.0123 – 0.8327	**0.044**	-0.0116	-0.0406 – 0.0173	0.431
	**Cephalexin**	ARIMA (0,1,1)	-0.5936	-1.0804 – -0.1068	**0.017**	0.0095	-0.0070 – 0.0260	0.258	(2,0,2) (1,0,0)[52]	-0.3128	-0.6073 – -0.0183	**0.037**	0.0096	-0.0012 – 0.0205	0.081
	**Clarithromycin**								ARIMA (2,1,3)	0.0058	-0.0934 – 0.1050	0.909	-0.0031	-0.0091 – 0.0029	0.310
	**Doxycycline**	ARIMA (1,1,1)	-0.1440	-0.6991 – 0.4111	0.611	-0.0024	-0.0325 – 0.0278	0.878	(1,1,1) (0,0,1)[52]	-0.1936	-0.3200 – -0.0672	**0.003**	-0.0074	-0.0130 – -0.0018	**0.009**
	**Fosfomycin**								(0,1,1) (1,0,0)[52]	0.0272	-0.2010 – 0.2554	0.815	-0.0059	-0.0156 – 0.0039	0.238
	**Nitrofurantoin**	ARIMA (2,0,2)	0.2995	0.0116 – 0.5874	**0.041**	-0.0034	-0.0142 – 0.0074	0.538	(0,1,1) (1,0,0)[52]	0.0070	-0.3376 – 0.3516	0.968	0.0130	-0.0009 – 0.0268	0.067
	**Penicillin**								ARIMA (0,1,1)	-0.0344	-0.0600 – -0.0088	**0,009**	0.0008	-0.0001 – 0.0017	0.074

Bold: p-value < 0.05

* Model parameters are displayed as 'SARIMA (p,d,q) (P,D,Q)S'. If no seasonality was present, the model is presented as: ARIMA(p,d,q)

[#] No results are shown for clarithromycin, fosfomycin and penicillin in Alberta to preserve subject anonymity, as the number of dispensations was on average less than 5 per week

LTCF: Long-term care facility; SARIMA: seasonal autoregressive integrated moving average; ARIMA: autoregressive integrated moving average; 95% CI: 95% Confidence interval;
ATC: Anatomical Therapeutic Chemical Classification

Supplementary Table S4. Sensitivity analysis 3 – Interrupted time series analysis showing the change in weekly oral antibiotic prescription rate per 1,000 LTCF residents after March 2020 in long-term care facilities in Alberta and Ontario, Canada; modeling a sudden increase of the step function in 3 weeks (week 10-12 of 2020) without a slope function

		Alberta				Ontario			
**Prescription rate category**		**Model parameters** *	**Step change**	**95% CI**	**p-value**	**Model parameters** *	**Step change**	**95% CI**	**p-value**
**Overall**		(0,1,2) (1,0,0)[52]	-3.7120	-6.4272 – -0.9967	**0.007**	(0,1,1) (0,1,1)[52]	-1.3006	-3.6284 – 1.0272	0.273
**Sex**	**Females**	(1,1,1) (0,0,1)[52]	-3.6399	-6.5166 – -0.7631	**0.013**	(0,1,1) (0,1,1)[52]	-1.4988	-4.0949 – 1.0972	0.258
	**Males**	(1,0,1) (1,0,0)[52]	-1.2934	-3.0050 – 0.4182	0.139	(0,1,1) (0,1,1)[52]	-1.1209	-3.3345 – 1.0926	0.3219
**Age**	**65-69**	ARIMA (0,0,0)	1.1191	-0.3590 – 2.5971	0.138	(1,0,2) (1,0,0)[52]	-1.7428	-4.2660 – 0.7805	0.176
	**70-74**	ARIMA (0,0,5)	-0.2851	-2.0444 – 1.4742	0.751	(0,1,1) (1,0,0)[52]	-2.1182	-4.4610 – 0.2246	0.076
	**75-79**	ARIMA (0,1,1)	-3.6619	-6.3022 – -1.0217	**0.007**	(0,1,2) (1,0,0)[52]	-3.5004	-6.0083 – -0,9926	**0.006**
	**80-84**	(0,1,1) (1,0,0)[52]	-3.6608	-6.6927 – -0.6288	**0.018**	(0,1,1) (0,1,1)[52]	-2.2434	-4.3442 – -0.1426	**0.036**
	**85-89**	ARIMA (1,0,1)	-1.9387	-3.7895 – -0.0879	**0.040**	(0,1,1) (1,0,0)[52]	-1.1780	-4.9916 – 2.6357	0.545
	**90+**	(1,0,1) (1,0,0)[52]	-0.9898	-2.6769 – 0.6972	0.250	(0,1,1) (0,1,1)[52]	-0.7554	-3.5464 – 2.0357	0.596
**ATC Class**	**J01A – Tetracyclines**	ARIMA (1,0,1)	-0.0341	-0.3700 – 0.3019	0.843	(1,1,1) (1,0,0)[52]	-0.2433	-0.4197 – -0.0669	**0.007**
	**J01C – Beta-lactams**	(1,1,2) (0,0,1)[52]	-1.0979	-1.8323 – -0.3634	**0.003**	(0,1,1) (0,1,1)[52]	-1.0751	-1.6816 – -0.4686	**<0.001**
	**J01D – Other beta-lactams**	ARIMA (0,1,1)	-0.6426	-1.2908 – 0.0056	0.052	(0,1,3) (1,0,0)[52]	-0.7134	-1.1646 – -0.2623	**0.002**
	**J01E – Sulfonamides and trimethoprim**	ARIMA (0,0,0)	-0.0382	-0.2076 – 0.1313	0.659	(0,1,1) (0,0,1)[52]	0.3204	0.0534 – 0.5874	**0.019**
	**J01F – Macrolides, lincosamides**	(1,0,1) (0,0,1)[52]	-0.1096	-0.2981 – 0.0788	0.254	(0,1,1) (1,0,0)[52]	0.3685	-0.3012 – 1.0381	0.281

	**and streptogramins**								
	**J01M – Quinolones**	(1,0,1) (0,0,1)[52]	-0.5630	-0.9244 – -0.2017	**0.002**	(0,1,1) (1,1,0)[52]	0.5486	-0.5575 – 1.6547	0.331
	**J01X – Other antibacterials**	ARIMA (0,0,2)	0.3018	0.1226 – 0.4810	**<0.001**	(1,0,1) (1,0,0)[52]	0.6276	0.3899 – 0.8653	**<0.001**
**Individual antibiotics**	**Amoxicillin**	(1,1,2) (0,0,1)[52]	-0.5526	-0.8708 – -0.2345	**<0.001**	(2,1,2) (0,0,1)[52]	-0.7520	-0.9297 – -0.5742	**<0.001**
	**Amoxicillin/ clavulanic acid**	ARIMA (1,0,1)	-0.2948	-0.6322 – 0.0425	0.087	(0,1,1) (1,1,0)[52]	-0.1943	-0.8039 – 0.4153	0.532
	**Azithromycin**	(1,0,1) (0,0,1)[52]	-0.0753	-0.2867 – 0.1360	0.485	(0,1,1) (0,1,1)[52]	0.6036	0.0778 – 1.1293	**0.024**
	**Cephalexin**	ARIMA (0,1,1)	-0.4210	-0.8828 – 0.0409	0.074	(2,0,2) (1,0,0)[52]	-0.1292	-0.3646 – 0.1062	0.282
	**Clarithromycin**					ARIMA (2,1,3)	-0.0209	-0.1313 – 0.0895	0.711
	**Doxycycline**	ARIMA (1,1,1)	-0.2583	-0.8930 – 0.3764	0.425	(1,1,1) (0,0,1)[52]	-0.2360	-0.4041 – -0.0678	**0.006**
	**Fosfomycin**					(0,1,1) (1,0,0)[52]	-0.0191	-0.2625 – 0.2244	0.878
	**Nitrofurantoin**	ARIMA (2,0,2)	0.2228	0.0652 – 0.3805	**0.006**	(0,1,1) (1,0,0)[52]	-0.1365	-0.5619 – 0.2889	0.529
	**Penicillin**					ARIMA (0,1,1)	-0.0162	-0.0362 – 0.0037	0.111

Bold: p-value < 0.05

* Model parameters are displayed as 'SARIMA (p,d,q) (P,D,Q)S'. If no seasonality was present, the model is presented as: ARIMA(p,d,q)

[#] No results are shown for clarithromycin, fosfomycin and penicillin in Alberta to preserve subject anonymity, as the number of dispensations was on average less than 5 per week

LTCF: Long-term care facility; SARIMA: seasonal autoregressive integrated moving average; ARIMA: autoregressive integrated moving average; 95% CI: 95% Confidence interval; ATC: Anatomical Therapeutic Chemical Classification
